# Dietary supplementation of valine, isoleucine, and tryptophan may overcome the negative effects of excess leucine in diets for weanling pigs containing corn fermented protein

**DOI:** 10.1186/s40104-024-01082-9

**Published:** 2024-09-10

**Authors:** Andrea P. Mallea, Charmaine D. Espinosa, Su A Lee, Minoy A. Cristobal, Leidy J. Torrez-Mendoza, Hans H. Stein

**Affiliations:** 1 Division of Nutritional Sciences, University of Illinois, Urbana, IL 61801 USA; 2 Department of Animal Sciences, University of Illinois, Urbana, IL 61801 USA; 3 Present Address: EnviroFlight, Raleigh, NC USA

**Keywords:** Branched-chain amino acids, Corn fermented protein, Leucine, Tryptophan, Valine, Weanling pigs

## Abstract

**Background:**

Diets with high inclusion of corn co-products such as corn fermented protein (CFP) may contain excess Leu, which has a negative impact on feed intake and growth performance of pigs due to increased catabolism of Val and Ile and reduced availability of Trp in the brain for serotonin synthesis. However, we hypothesized that the negative effect of using CFP in diets for weanling pigs may be overcome if diets are fortified with crystalline sources of Val, Trp, and (or) Ile.

**Methods:**

Three hundred and twenty weanling pigs were randomly allotted to one of 10 dietary treatments in a completely randomized design, with 4 pigs per pen and 8 replicate pens per treatment. A corn-soybean meal diet and 2 basal diets based on corn and 10% CFP or corn and 20% CFP were formulated. Seven additional diets were formulated by fortifying the basal diet with 20% CFP with Ile, Trp, Val, Ile and Val, Ile and Trp, Trp and Val, or Ile, Trp and Val. A two-phase feeding program was used, with d 1 to 14 being phase 1 and d 15 to 28 being phase 2. Fecal scores were recorded every other day. Blood samples were collected on d 14 and 28 from one pig per pen. On d 14, fecal samples were collected from one pig per pen in 3 of the 10 treatments to determine volatile fatty acids, ammonium concentration, and microbial protein. These pigs were also euthanized and ileal tissue was collected.

**Results:**

There were no effects of dietary treatments on any of the parameters evaluated in phase 1. Inclusion of 10% or 20% CFP in diets reduced (*P* < 0.05) final body weight on d 28, and average daily gain (ADG) and average daily feed intake (ADFI) in phase 2 and for the entire experimental period. However, pigs fed the CFP diet supplemented with Val, Ile, and Trp had final body weight, ADFI, ADG and gain to feed ratio in phase 2 and for the entire experiment that was not different from pigs fed the control diet. Fecal scores in phase 2 were reduced (*P* < 0.05) if CFP was used.

**Conclusions:**

Corn fermented protein may be included by up to 20% in diets for weanling pigs without affecting growth performance, gut health, or hindgut fermentation, if diets are fortified with extra Val, Trp, and Ile. Inclusion of CFP also improved fecal consistency of pigs.

## Background

High protein corn co-products such as corn fermented protein (CFP) have been developed in recent years, and most of these ingredients are produced by fractionating corn co-products from the dry grind ethanol industry [1]. Corn fermented protein is a product containing fermented fiber and protein and spent yeast. The CFP contains approximately 50% crude protein (CP). The yeast content in CFP is 20% to 29% [1]. This proportion is greater than the estimated 7% to 10% of spent yeast in conventional distillers dried grains with solubles [1, 2]. Therefore, spent yeast contributes around 25% of the total protein.

Corn fermented protein has a greater amino acid (AA) digestibility than conventional corn distillers dried grains with solubles, and therefore, may be used in diets for pigs as a source of AA [3]. Indeed, it is possible to formulate diets for pigs based on corn and CFP, but such diets may contain more than twice as much Leu than required by pigs. However, there is a negative relationship between dietary Leu and brain synthesis of serotonin, which results in reduced feed intake of pigs fed diets containing excess Leu [4, 5]. Pigs fed diets with elevated levels of Leu also have a reduced protein synthesis because of increased Val and Ile metabolism caused by the excess dietary Leu [6]. As a consequence, pigs often have reduced growth performance if fed diets with CFP inclusion levels greater than 15%. However, it may be possible to correct the Leu imbalance in CFP diets by adding crystalline sources of Val, Trp, and/or Ile to the diets, and it may, therefore, be possible to include CFP in diets for weanling pigs without influencing growth performance or intestinal health. However, at this time, limited research has been conducted to confirm this hypothesis. Therefore, the hypothesis that the negative effect on pig growth performance of using CFP in diets for weanling pigs may be mitigated if diets contain Val, Trp, and (or) Ile above current requirement estimates was tested.

## Methods

The protocol for the experiment was submitted to the Institutional Animal Care and Use Committee at the University of Illinois and was approved before initiation of the experiment. Pigs that were the offspring of Line 800 boars mated to Camborough females were used (Pig Improvement Company, Hendersonville, TN, USA). The CFP used in the experiment was sourced from Green Plains Energy (Omaha, NE, USA; Table 1), and soybean meal (SBM) was sourced from Stutzman’s Feed & Supply (Arthur, IL, USA). Ground yellow corn was obtained from the University of Illinois Feed Mill (Urbana, IL, USA).

**Table 1:** Analyzed composition of main ingredients (as-fed basis)^1^

Item	Soybean meal	Corn fermented protein	Corn
Gross energy, kcal/kg	4,194	4,950	3,847
Crude protein, %	46.98	50.26	6.72
Dry matter, %	89.35	91.72	86.41
Ash, %	12.73	9.34	1.95
Acid hydrolyzed ether extract, %	2.86	5.82	3.53
Insoluble dietary fiber, %	16.50	24.20	10.20
Soluble dietary fiber, %	2.90	2.50	-
Total dietary fiber, %	19.40	26.70	10.20
Indispensable amino acids, %			
Arg	3.26	2.30	0.29
His	1.35	1.36	0.18
Ile	2.24	2.34	0.24
Leu	3.55	6.51	0.69
Lys	2.66	1.80	0.22
Met	0.62	1.11	0.12
Phe	2.36	2.92	0.30
Thr	1.82	1.96	0.22
Trp	0.62	0.49	0.05
Val	2.25	2.84	0.33
Dispensable amino acids, %			
Ala	2.00	3.81	0.44
Asp	5.25	3.62	0.42
Cys	0.66	0.93	0.14
Glu	8.33	8.52	1.11
Gly	1.95	2.11	0.27
Pro	2.25	3.99	0.52
Ser	2.13	2.22	0.27
Tyr	1.70	2.19	0.15

^1^Corn fermented protein was sourced from Green Plains Energy, Omaha, NE, USA

### Diets, animals, and experimental design

A two-phase feeding program was used with d 1 to 14 as phase 1 and d 15 to 28 as phase 2. A total of 320 weanling pigs with an initial body weight (BW) of 6.11 ± 0.66 kg were used in 2 blocks of 160 pigs. Within each block, pigs were randomly assigned to 10 dietary treatments in a randomized complete block design. There were 2 barrows and 2 gilts in each pen and 8 replicate pens per treatment. A control diet based on corn and SBM was formulated and 2 basal diets were formulated based on corn and 10% CFP or corn and 20% CFP (Tables 2 and 3). Seven additional diets were formulated by supplementing the diet containing 20% CFP with crystalline Ile, Trp, and Val as follows: 1) + 0.10% Ile; 2) + 0.05% Trp; 3) + 0.10% Val; 4) + 0.10% Ile + 0.10% Val; 5) + 0.10% Ile + 0.05% Trp; 6) + 0.10% Val + 0.05% Trp; and 7) + 0.10% Ile + 0.10% Val + 0.05% Trp. The inclusion levels of Val, Ile, and Trp were based on previous data demonstrating that these levels of the AA ameliorate negative effects of excess Leu in diets for growing pigs [7]. All diets were formulated to meet current requirement estimates for AA, vitamins, and minerals [8].

**Table 2:** Composition of phase 1 experimental diets (as-fed basis)

Ingredient, %	Control diet	10% CFP^1^	20% CFP	CFP + Ile	CFP + Trp	CFP + Val	CFP + Ile + Val	CFP + Ile + Trp	CFP + Val + Trp	CFP + Ile + Val + Trp
Ground corn	44.70	47.25	48.89	48.79	48.84	48.79	48.69	48.74	48.74	48.64
Soybean meal	20.00	10.00	-	-	-	-	-	-	-	-
Corn fermented protein	-	10.00	20.00	20.00	20.00	20.00	20.00	20.00	20.00	20.00
Soybean oil	2.80	2.80	2.80	2.80	2.80	2.80	2.80	2.80	2.80	2.80
Whey powder	20.00	20.00	20.00	20.00	20.00	20.00	20.00	20.00	20.00	20.00
ESBM^1^	9.00	6.00	4.00	4.00	4.00	4.00	4.00	4.00	4.00	4.00
Limestone	1.04	1.18	1.31	1.31	1.31	1.31	1.31	1.31	1.31	1.31
Dicalcium phosphate	0.70	0.65	0.60	0.60	0.60	0.60	0.60	0.60	0.60	0.60
L-Lysine	0.47	0.74	0.96	0.96	0.96	0.96	0.96	0.96	0.96	0.96
DL-Methionine	0.23	0.18	0.17	0.17	0.17	0.17	0.17	0.17	0.17	0.17
L-Threonine	0.13	0.17	0.20	0.20	0.20	0.20	0.20	0.20	0.20	0.20
L-Tryptophan	-	0.04	0.07	0.07	0.12	0.07	0.07	0.12	0.12	0.12
L-Valine	0.03	0.04	0.03	0.03	0.03	0.13	0.13	0.03	0.13	0.13
L-Isoleucine	-	-	-	0.10	-	-	0.10	0.10	-	0.10
L-Histidine	-	0.05	0.07	0.07	0.07	0.07	0.07	0.07	0.07	0.07
Salt	0.40	0.40	0.40	0.40	0.40	0.40	0.40	0.40	0.40	0.40
Vitamin-mineral premix^2^	0.50	0.50	0.50	0.50	0.50	0.50	0.50	0.50	0.50	0.50
Calculated values										
ME, kcal/kg	3,456	3,443	3,439	3,436	3,438	3,436	3,433	3,434	3,434	3,431
Crude protein, %	19.26	18.42	18.10	18.09	18.10	18.09	18.09	18.09	18.09	18.08
Total Ca, %	0.80	0.80	0.80	0.80	0.80	0.80	0.80	0.80	0.80	0.80
P^3^, %	0.40	0.40	0.40	0.40	0.40	0.40	0.40	0.40	0.40	0.40
Amino acids^4^, %										
Arg	1.13	0.92	0.74	0.74	0.74	0.74	0.74	0.74	0.74	0.74
His	0.46	0.46	0.46	0.46	0.46	0.46	0.46	0.46	0.46	0.46
Ile	0.79	0.73	0.69	0.79	0.69	0.69	0.79	0.79	0.69	0.79
Leu	1.45	1.63	1.84	1.84	1.84	1.84	1.84	1.84	1.84	1.84
Lys	1.40	1.40	1.40	1.40	1.40	1.40	1.40	1.40	1.40	1.40
Met	0.48	0.45	0.47	0.47	0.47	0.47	0.47	0.47	0.47	0.47
Met + Cys	0.74	0.74	0.74	0.74	0.74	0.74	0.74	0.74	0.74	0.74
Phe	0.82	0.80	0.80	0.80	0.80	0.80	0.80	0.80	0.80	0.80
Thr	0.80	0.80	0.80	0.80	0.80	0.80	0.80	0.80	0.80	0.80
Trp	0.23	0.23	0.23	0.23	0.28	0.23	0.23	0.28	0.28	0.28
Val	0.86	0.86	0.86	0.86	0.86	0.96	0.96	0.86	0.96	0.96

^1^ESBM, enzyme-treated soybean meal (Hamlet Protein, Findley, OH, USA); CFP, corn fermented protein (Green Plains Energy, Omaha, NE, USA)

^2^The vitamin-mineral premix provided the following quantities of vitamins and micro-minerals per kilogram of complete diet: vitamin A as retinyl acetate, 11,150 IU; vitamin D_3_ as cholecalciferol, 2,210 IU; vitamin E as DL-alpha tocopheryl acetate, 66 IU; vitamin K as menadione nicotinamide bisulfate, 1.42 mg; thiamin as thiamine mononitrate, 1.10 mg; riboflavin, 6.59 mg; pyridoxine as pyridoxine hydrochloride, 1.00 mg; vitamin B_12_, 0.03 mg; D-pantothenic acid as D-calcium pantothenate, 23.6 mg; niacin, 44.1 mg; folic acid, 1.59 mg; biotin, 0.44 mg; Cu, 20 mg as copper chloride; Fe, 125 mg as iron sulfate; I, 1.26 mg as ethylenediamine dihydriodide; Mn, 60.2 mg as manganese hydroxychloride; Se, 0.30 mg as sodium selenite and selenium yeast; and Zn, 125.1 mg as zinc hydroxychloride

^3^Standardized total tract digestible P

^4^Amino acids are indicated as standardized ileal digestible amino acids

**Table 3:** Composition of phase 2 experimental diets containing corn fermented protein (CFP) (as-fed basis)^1^

Ingredient, %	Control diet	10% CFP	20% CFP	CFP + Ile	CFP + Trp	CFP + Val	CFP + Ile + Val	CFP + Ile + Trp	CFP + Val + Trp	CFP + Ile + Val + Trp
Ground corn	54.49	54.38	56.97	56.87	56.92	56.87	56.77	56.82	56.82	56.72
Soybean meal	29.00	19.00	6.00	6.00	6.00	6.00	6.00	6.00	6.00	6.00
Corn fermented protein	-	10.00	20.00	20.00	20.00	20.00	20.00	20.00	20.00	20.00
Soybean oil	2.80	2.80	2.80	2.80	2.80	2.80	2.80	2.80	2.80	2.80
Whey powder	10.00	10.00	10.00	10.00	10.00	10.00	10.00	10.00	10.00	10.00
Limestone	0.95	1.10	1.25	1.25	1.25	1.25	1.25	1.25	1.25	1.25
Dicalcium phosphate	0.88	0.77	0.73	0.73	0.73	0.73	0.73	0.73	0.73	0.73
L-Lysine	0.50	0.65	0.88	0.88	0.88	0.88	0.88	0.88	0.88	0.88
DL-Methionine	0.23	0.18	0.15	0.15	0.15	0.15	0.15	0.15	0.15	0.15
L-Threonine	0.17	0.16	0.19	0.19	0.19	0.19	0.19	0.19	0.19	0.19
L-Tryptophan	-	0.02	0.06	0.06	0.11	0.06	0.06	0.11	0.11	0.11
L-Valine	0.08	0.02	0.02	0.02	0.02	0.12	0.12	0.02	0.12	0.12
L-Isoleucine	-	-	-	0.10	-	-	0.10	0.10	-	0.10
L-Histidine	-	0.02	0.05	0.05	0.05	0.05	0.05	0.05	0.05	0.05
Salt	0.40	0.40	0.40	0.40	0.40	0.40	0.40	0.40	0.40	0.40
Vitamin-mineral premix^2^	0.50	0.50	0.50	0.50	0.50	0.50	0.50	0.50	0.50	0.50
Calculated values										
ME, kcal/kg	3,382	3,399	3,409	3,406	3,408	3,406	3,402	3,404	3,404	3,401
Crude protein, %	17.68	18.38	17.89	17.89	17.89	17.89	17.88	17.88	17.88	17.88
Total Ca, %	0.75	0.75	0.75	0.75	0.75	0.75	0.75	0.75	0.75	0.75
P^3^, %	0.36	0.36	0.36	0.36	0.36	0.36	0.36	0.36	0.36	0.36
Amino acids^4^, %										
Arg	1.06	0.96	0.77	0.77	0.77	0.77	0.77	0.77	0.77	0.77
His	0.44	0.44	0.44	0.44	0.44	0.44	0.44	0.44	0.44	0.44
Ile	0.69	0.70	0.66	0.76	0.66	0.66	0.76	0.76	0.66	0.76
Leu	1.31	1.60	1.80	1.80	1.80	1.80	1.80	1.80	1.80	1.80
Lys	1.29	1.29	1.29	1.29	1.29	1.29	1.29	1.29	1.29	1.29
Met	0.46	0.45	0.45	0.45	0.45	0.45	0.45	0.45	0.45	0.45
Met + Cys	0.71	0.71	0.71	0.71	0.71	0.71	0.71	0.71	0.71	0.71
Phe	0.75	0.80	0.80	0.80	0.80	0.80	0.80	0.80	0.80	0.80
Thr	0.76	0.76	0.76	0.76	0.76	0.76	0.76	0.76	0.76	0.76
Trp	0.21	0.21	0.21	0.21	0.26	0.21	0.21	0.26	0.26	0.26
Val	0.82	0.82	0.82	0.82	0.82	0.92	0.92	0.82	0.92	0.92

^1^The corn fermented protein was sourced from Green Plains Energy, Omaha, NE, USA

^2^The vitamin-mineral premix provided the following quantities of vitamins and micro-minerals per kilogram of complete diet: vitamin A as retinyl acetate, 11,150 IU; vitamin D_3_ as cholecalciferol, 2,210 IU; vitamin E as DL-alpha tocopheryl acetate, 66 IU; vitamin K as menadione nicotinamide bisulfate, 1.42 mg; thiamin as thiamine mononitrate, 1.10 mg; riboflavin, 6.59 mg; pyridoxine as pyridoxine hydrochloride, 1.00 mg; vitamin B_12_, 0.03 mg; D-pantothenic acid as D-calcium pantothenate, 23.6 mg; niacin, 44.1 mg; folic acid, 1.59 mg; biotin, 0.44 mg; Cu, 20 mg as copper chloride; Fe, 125 mg as iron sulfate; I, 1.26 mg as ethylenediamine dihydriodide; Mn, 60.2 mg as manganese hydroxychloride; Se, 0.30 mg as sodium selenite and selenium yeast; and Zn, 125.1 mg as zinc hydroxychloride

^3^Standardized total tract digestible P

^4^Amino acids are indicated as standardized ileal digestible amino acids

Pigs were housed in pens with fully slatted plastic floors. Each pen was equipped with a feeder and a nipple drinker, and pigs had free access to feed and water throughout the experiment.

### Sample and data collection

Individual pig weight was recorded at the beginning of the experiment, on d 14, and at the end of the 28-day experiment. Daily feed allotments were recorded and the weight of feed left in the feeders was recorded on d 14 and on the last day of the experiment to calculate feed consumption. Fecal scores were assessed visually per pen every other day using a score from 1 to 5 (1 = normal feces; 2 = moist feces; 3 = mild diarrhea; 4 = severe diarrhea; and 5 = watery diarrhea).

On d 14 and on the last day of the experiment, 2 blood samples were collected from one pig in each pen that had a BW closest to the pen average. One sample was collected in heparinized vacutainers, and the other sample was collected in vacutainers containing EDTA. Blood samples were centrifuged at 4,000 × *g* at 4 °C for 13 min, and plasma was collected and stored at –20 ºC until analysis. Heparinized samples were analyzed for blood urea nitrogen, total protein, and albumin using a Beckman Coulter Clinical Chemistry AU analyzer at the University of Illinois Veterinary Diagnostic Laboratory. Samples treated with EDTA were analyzed for peptide YY and immunoglobulin G using ELISA kits according to the recommendations from the manufacturer (Phoenix Pharmaceuticals Inc., Burlingame, CA, USA; Bethyl Laboratories Inc., Montgomery, TX, USA, respectively).

On d 14, one pig per pen (the pig with a BW closest to the pen average) in 3 of the 10 dietary treatments (i.e., corn-SBM basal diet and the 2 basal diets containing 10% or 20% CFP) was euthanized via captive bolt penetration. Ileal tissue samples between 2 and 3 cm were collected approximately 80 cm from the ileal-cecal junction. Samples were cut and pinned with the serosa side down on a piece of cardboard. Samples were then fixed in 10% neutral buffered formalin until processing for immunohistochemistry staining and morphological evaluation. After fixation, all tissue samples were sectioned and transferred to slides. Villus height was measured from the villus tip to the base, and crypt depth was measured from the crypt-villus junction to the base of the crypt. Lamina propria thickness were also measured at the midpoint of the villus. Villus height:crypt depth ratio was also calculated.

Fecal samples from these pigs were also collected and analyzed for microbial protein, fecal NH_3,_ and volatile fatty acids (VFA). For microbial protein, 5 to 10 g of feces were collected and stored at –20 °C until analyzed. For VFA and NH_3_ analysis, 5 g of feces were placed in 15-mL tubes and samples were stabilized in 2 mol/L HCl and stored at –20 °C until analyzed. Fecal ammonia concentrations were determined according to the method by Chaney and Marbach [9] and VFA were determined using previously established procedures [10].

Fecal samples were fractionated using differential centrifugation [11]. Samples were then centrifuged at 250 relative centrifugal force for 15 min at 4 °C, which separated fractions that contained undigested feed particles in the precipitate and porcine cells in the supernatant [12]. The supernatant was centrifuged at 14,500 relative centrifugal force for 30 min at 4 °C, which resulted in a precipitate that contained microbial cells [12]. This precipitate was then subjected to a lysis buffer, which contained 100 mmol/L of tris(hydroxymethyl)aminomethane at pH 7.2, 0.5% sodium dodecyl sulfate, and 0.5% sodium deoxycholate. Protein concentration of the lysed microbial cells was analyzed using Pierce Bicinchoninic Acid Assay kit (ThermoFisher Scientific, Waltham, MA, USA).

### Chemical analysis

All diet and ingredient samples were analyzed in duplicate for concentrations of gross energy using a bomb calorimeter (Model 6400, Parr Instruments, Moline, IL, USA), and N was analyzed by combustion (method 990.03; [13]) using a LECO FP628 analyzer (LECO Corp., Saint Joseph, MI, USA) with the subsequent calculation of crude protein as N × 6.25 (Tables 3, 4 and 5). Dry matter was also analyzed in diet and ingredient samples by oven drying at 135 °C for 2 h (method 930.15; [13]) and these samples were also analyzed for dry ash (method 942.05; [13]). All diet and ingredient samples were analyzed for acid hydrolyzed ether extract using the acid hydrolysis filter bag technique (Ankom HCl Hydrolysis System; Ankom Technology, Macedon, NY, USA) followed by crude fat extraction using petroleum ether (AnkomXT15 Extractor; Ankom Technology, Macedon, NY, USA). All diet and ingredient samples were also analyzed for AA (method 982.30 E (a, b, c); [13]). Ingredients were also analyzed for insoluble dietary fiber and soluble dietary fiber according to method 991.43 [13] using the AnkomTDF Dietary Fiber Analyzer (Ankom Technology, Macedon, NY, USA). Total dietary fiber was calculated as the sum of insoluble and soluble dietary fiber.

**Table 4:** Analyzed nutrient composition of phase 1 diets

Item	Control diet	10% CFP^1^	20% CFP	CFP + Ile	CFP + Trp	CFP + Val	CFP + Ile + Val	CFP + Ile + Trp	CFP + Val + Trp	CFP + Ile + Val + Trp
Gross energy, kcal/kg	3,998	4,010	4,001	3,989	4,005	3,994	4,071	4,024	4,063	3,976
Crude protein, %	21.35	20.45	20.18	20.79	21.48	20.18	20.63	20.27	20.43	20.24
Dry matter, %	89.83	89.49	89.92	89.98	89.78	89.86	89.94	89.68	89.91	89.83
Ash, %	9.85	8.41	8.19	8.26	8.87	8.16	8.64	8.20	8.36	8.74
Acid hydrolyzed ether extract, %	4.59	4.63	4.74	4.41	4.85	4.25	4.16	4.34	4.95	4.77
Indispensable amino acids, %										
Arg	1.24	0.99	0.79	0.82	0.80	0.79	0.82	0.80	0.79	0.81
His	0.52	0.51	0.51	0.52	0.52	0.50	0.51	0.51	0.50	0.52
Ile	0.97	0.87	0.79	0.89	0.79	0.79	0.88	0.89	0.77	0.88
Leu	1.69	1.83	2.01	2.05	2.05	2.03	2.05	2.03	2.04	2.05
Lys	1.61	1.62	1.62	1.61	1.59	1.58	1.58	1.60	1.59	1.58
Met	0.4	0.45	0.45	0.45	0.47	0.44	0.47	0.45	0.45	0.45
Phe	0.97	0.92	0.88	0.89	0.89	0.87	0.89	0.89	0.86	0.86
Thr	0.91	0.90	0.92	0.93	0.93	0.90	0.91	0.89	0.90	0.93
Trp	0.24	0.24	0.24	0.24	0.27	0.24	0.24	0.28	0.28	0.27
Val	1.02	1.01	1.00	1.01	1.01	1.10	1.11	1.00	1.10	1.10
Dispensable amino acids, %										
Ala	0.95	1.07	1.20	1.24	1.22	1.18	1.22	1.20	1.20	1.22
Asp	2.13	1.74	1.42	1.46	1.43	1.40	1.44	1.43	1.40	1.44
Cys	0.34	0.34	0.35	0.35	0.35	0.34	0.35	0.35	0.34	0.34
Glu	3.62	3.31	3.10	3.18	3.15	3.05	3.16	3.09	3.01	3.15
Gly	0.79	0.73	0.68	0.70	0.69	0.67	0.68	0.67	0.67	0.69
Pro	1.14	1.22	1.33	1.39	1.37	1.33	1.36	1.35	1.32	1.36
Ser	0.84	0.79	0.76	0.79	0.78	0.76	0.78	0.78	0.76	0.78

^1^CFP, corn fermented protein (Green Plains Energy, Omaha, NE, USA)

**Table 5:** Analyzed nutrient composition of phase 2 diets

Item	Control diet	10% CFP^1^	20% CFP	CFP + Ile	CFP + Trp	CFP + Val	CFP + Ile + Val	CFP + Ile + Trp	CFP + Val + Trp	CFP + Ile + Val + Trp
Gross energy, kcal/kg	4,004	4,059	4,087	4,105	4,136	4,104	4,091	4,047	4,112	4,103
Crude protein, %	19.21	19.90	19.18	20.01	19.42	20.81	20.26	20.54	19.55	19.99
Dry matter, %	89.87	89.70	89.89	89.91	89.97	89.84	90.04	89.65	89.10	89.66
Ash, %	10.04	9.37	8.42	9.13	8.61	8.11	8.85	8.59	8.24	8.86
Acid hydrolyzed ether extract, %	4.14	4.69	4.91	4.42	4.14	4.76	4.87	4.65	4.11	4.18
Indispensable amino acids, %										
Arg	1.12	1.08	0.88	0.88	0.87	0.82	0.89	0.89	0.89	0.84
His	0.50	0.51	0.51	0.50	0.54	0.54	0.52	0.54	0.52	0.53
Ile	0.90	0.90	0.82	0.93	0.83	0.83	0.95	0.95	0.85	0.94
Leu	1.63	1.90	2.02	2.04	2.02	2.05	2.09	2.11	2.04	2.08
Lys	1.53	1.52	1.53	1.40	1.47	1.52	1.51	1.44	1.44	1.48
Met	1.42	1.50	1.42	1.42	1.45	1.50	1.50	1.44	1.44	1.44
Phe	0.95	0.95	0.96	0.92	0.91	0.95	0.95	0.98	0.96	0.97
Thr	0.88	0.87	0.86	0.89	0.89	0.89	0.85	0.89	0.89	0.88
Trp	0.22	0.23	0.23	0.23	0.28	0.23	0.23	0.28	0.28	0.28
Val	1.01	1.01	1.03	1.01	1.03	1.13	1.15	1.02	1.17	1.15
Dispensable amino acids, %										
Ala	0.93	1.20	1.20	1.20	1.21	1.24	1.25	1.28	1.27	1.28
Asp	1.93	1.79	1.46	1.47	1.44	1.54	1.50	1.53	1.53	1.51
Cys	0.29	0.33	0.31	0.33	0.32	0.35	0.34	0.36	0.36	0.35
Glu	3.41	3.42	3.15	3.11	3.13	3.25	3.21	3.33	3.28	3.31
Gly	0.75	0.77	0.71	0.71	0.71	0.74	0.72	0.74	0.73	0.74
Pro	1.09	1.26	1.33	1.32	1.38	1.33	1.35	1.36	1.34	1.35
Ser	0.79	0.80	0.77	0.78	0.78	0.80	0.79	0.80	0.80	0.80

^1^CFP, corn fermented protein (Green Plains Energy, Omaha, NE, USA)

### Statistical analysis

Data for growth performance were summarized to calculate average daily gain (ADG), average daily feed intake (ADFI), and gain to feed ratio (G:F) for each pen and for each treatment group. Normality of data was verified and outliers were identified using the UNIVARIATE procedure of SAS. Data for growth performance were analyzed using the PROC MIXED of SAS with the experimental unit being the pen, whereas pig was the experimental unit in the analyses of other response parameters. The model included diet as fixed effect and block and replicate within block as random effects. Treatment means were calculated using the LSMEANS statement and means were separated using the pdiff option with the Tukeys adjustment if the model was significant. Statistical significance was considered at *P* < 0.05.

## Results

All animals remained healthy throughout the experiment and readily consumed their assigned diets. There were no effects of dietary treatments on any of the parameters evaluated in phase 1. Inclusion of 10% or 20% CFP in diets without addition of extra AA reduced (*P* < 0.05) final BW on d 28 and also reduced (*P* < 0.05) ADG and ADFI in phase 2 and for the entire experimental period (Table 6). Pigs fed the diet containing 20% CFP supplemented with all three AA had a greater (*P* < 0.05) final BW than pigs fed the two basal diets or diets supplemented with only Trp, only Ile, or Ile and Trp. Pigs fed the diet containing 20% CFP supplemented with all three AA also had a greater (*P* < 0.05) ADG in phase 2 and for the overall experiment than pigs fed the basal diets or diets supplemented with only Ile, only Trp, Ile and Val, or Ile and Trp.

**Table 6:** Growth performance of pigs fed experimental diets^1,2,3,4^

Item	Control diet	10% CFP	20% CFP	CFP + Ile	CFP + Trp	CFP + Val	CFP + Ile + Val	CFP + Ile + Trp	CFP + Val + Trp	CFP + Ile + Val + Trp	SEM	*P* value
Body weight, kg												
d 1	6.33	6.29	6.34	6.33	6.32	6.30	6.29	6.31	6.31	6.29	0.23	0.154
d 14	7.42	7.26	7.35	7.21	7.20	7.35	7.34	6.97	7.40	7.43	0.28	0.398
d 28	14.22^a^	12.58^cde^	12.07^cdef^	11.80^ef^	11.93^def^	13.02^bcd^	13.08^bc^	11.44^f^	13.00^bcde^	13.77^ab^	0.54	0.001
ADG, g												
d 1 to 14	78	69	72	62	63	74	75	47	78	81	9.4	0.281
d 15 to 28	485^a^	379^ cd^	337^d^	334^d^	337^d^	405^bc^	375^ cd^	319^d^	399^bc^	453^ab^	23.7	0.001
d 1 to 28	281^a^	224^cde^	204^cdef^	198^ef^	200^def^	240^bc^	225^cde^	183^f^	238^bcd^	267^ab^	14.3	0.001
ADFI, g												
d 1 to 14	141	136	135	128	135	141	136	118	146	157	10.3	0.080
d 15 to 28	662^a^	549^ cd^	503^cde^	493^de^	499^cde^	572^bcd^	549^ cd^	441^e^	577^bc^	646^ab^	31.84	0.001
d 1 to 28	401^a^	343^b^	319^bc^	313^bc^	317^bc^	356^ab^	349^b^	280^c^	361^ab^	402^a^	18.76	0.001
G:F												
d 1 to 14	0.55	0.44	0.52	0.44	0.45	0.51	0.49	0.35	0.52	0.51	0.05	0.119
d 15 to 28	0.73	0.69	0.66	0.68	0.67	0.70	0.68	0.73	0.69	0.70	0.37	0.349
d 1 to 28	0.70	0.65	0.63	0.63	0.62	0.67	0.64	0.65	0.66	0.66	0.01	0.154

^1^CFP, corn fermented protein (Green Plains Energy, Omaha, NE, USA)

^2^Data are least square means of 8 observations per treatment

^3^ADFI, average daily feed intake; ADG, average daily gain; G:F, gain to feed ratio

^4^All pigs were fed phase 1 diets for 14 d post-weaning, and they were then fed phase 2 diets from d 15 to 28 post-weaning

^a^^−^^f^Within a row, means not sharing a common superscript letter are different (*P* < 0.05)

There were no differences between pigs fed the control diet and pigs fed the diet with three AA for final BW and ADG in phase 2 or for the entire experiment. However, pigs fed the diet containing 20% CFP and Val, Trp, and Ile had greater (*P* < 0.05) ADFI in phase 2 and for the entire experiment than pigs fed the basal diets or diets supplemented with only Ile, only Trp, Ile and Val, or Ile and Trp.

From d 15 to 28, fecal scores were reduced (*P* < 0.05) if CFP was included in the diet (Table 7), and for the entire experimental period, fecal scores were reduced (*P* < 0.05) if 20% CFP was included in the diet except for the diet supplemented with Ile and Val. No differences among experimental diets were observed for blood urea N, total protein, albumin, peptide YY, or immunoglobulin G on d 14 (Table 8). However, on d 28, pigs fed the diet with 20% CFP and only Val, only Ile, Val and Trp, or Val, Trp, and Ile had reduced (*P* < 0.01) blood urea N compared with pigs fed the control diet. No differences in villus height, crypth depth, or villus height:crypt depth ratio of pigs were observed among pigs fed the control diet or the 2 basal diets containing CFP (Table 9). Inclusion of CFP in experimental diets did also not affect the concentration of microbial protein in feces of pigs (Table 10). Likewise, concentrations of VFA and ammonium in feces were not affected by inclusion of CFP in the diets.

**Table 7:** Fecal scores of pigs fed the experimental diets^1,2,3^

Item	Control diet	10% CFP	20% CFP	CFP + Ile	CFP + Trp	CFP + Val	CFP + Ile + Val	CFP + Ile + Trp	CFP + Val + Trp	CFP + Ile + Val + Trp	SEM	*P* value
d 1 to 14	2.42	2.25	2.12	2.03	2.03	2.17	2.51	2.08	2.21	2.11	0.21	0.295
d 15 to 28	1.67^a^	1.44^b^	1.23^c^	1.23^c^	1.37^bc^	1.21^c^	1.26^bc^	1.26^bc^	1.21^c^	1.33^bc^	0.13	0.001
d 1 to 28	2.05^a^	1.84^abc^	1.67^bc^	1.63^c^	1.70^bc^	1.75^bc^	1.89^ab^	1.67^bc^	1.71^bc^	1.72^bc^	0.14	0.030

^1^CFP, corn fermented protein (Green Plains Energy, Omaha, NE, USA)

^2^Data are least square means of 8 observations per treatment

^3^Fecal scores were visually assessed every other day by one independent observer for 28 d. Fecal score: 1, normal feces; 2, moist feces; 3, mild diarrhea; 4, severe diarrhea; and 5, watery diarrhea

^a^^−^^c^Within a row, means not sharing a common superscript letter are different (*P* < 0.05)

**Table 8:** Blood characteristics on d 14 and 28 of pigs fed experimental diets^1,2^

Item	Control diet	10% CFP	20% CFP	CFP + Ile	CFP + Trp	CFP + Val	CFP + Ile + Val	CFP + Ile + Trp	CFP + Val + Trp	CFP + Ile + Val + Trp	SEM	*P* value
d 14												
Blood urea nitrogen, mg/dL	8.71	6.75	6.87	6.00	6.12	5.12	5.75	6.54	4.50	5.37	1.06	0.371
Total protein, mg/dL	4.53	4.50	4.31	4.38	4.47	4.42	4.38	4.40	4.41	4.38	0.11	0.858
Albumin, mg/dL	2.71	2.73	2.72	2.66	2.67	2.63	2.56	2.68	2.57	2.66	0.09	0.611
Peptide YY, ng/mL	1.93	1.81	1.73	1.76	1.84	1.84	1.86	1.72	1.84	1.89	0.12	0.960
Immunoglobulin G, mg/mL	5.35	3.71	3.92	4.78	3.07	3.17	3.05	4.06	4.29	3.60	0.84	0.290
d 28												
Blood urea nitrogen, mg/dL	6.25^a^	4.62^abc^	4.50^abc^	4.00^bcd^	5.00^abc^	3.62^ cd^	4.62^abc^	5.62^ab^	2.59^d^	3.37^ cd^	0.66	0.006
Total protein, mg/dL	4.82	4.86	4.68	4.71	4.75	4.83	4.60	4.66	4.62	4.80	0.10	0.390
Albumin, mg/dL	3.18^a^	3.12^ab^	2.97^abcde^	2.85^cde^	2.83^de^	3.03^abcd^	2.72^e^	2.86^bcde^	2.82^de^	3.11^abc^	0.09	0.009
Peptide YY, ng/mL	2.69	2.72	2.79	2.75	2.72	2.96	2.96	3.16	2.95	2.94	1.03	0.971
Immunoglobulin G, mg/mL	6.59	4.59	4.61	5.64	4.93	4.59	4.68	4.70	4.71	3.91	0.91	0.679

^1^CFP, corn fermented protein (Green Plains Energy, Omaha, NE, USA)

^2^Data are least square means of 8 observations per treatment

^a^^−^^e^Within a row, means not sharing a common superscript letter are different (*P* < 0.05)

**Table 9:** Morphology measurements of ileal samples on d 14 of pigs fed the control diet or one of the two basal diets^1,2^

Item	Control diet	10% CFP	20% CFP	SEM	*P* value
Villus height, μm	283.15	260.95	285.18	35.13	0.866
Crypt depth, μm	241.28	224.23	206.91	11.17	0.119
Villus height:crypt depth ratio	1.24	1.33	1.50	0.19	0.534
Lamina propria thickness, μm	67.72	62.49	55.96	5.14	0.337

^1^CFP, corn fermented protein (Green Plains Energy, Omaha, NE, USA)

^2^Data are least square means of 8 observations per treatment

**Table 10:** Intestinal microbial protein concentrations (mg/g, dry matter), rate of fermentation of volatile fatty acids, and ammonia concentration in feces on d 14 of pigs fed the control diet or one of the two basal diets^1,2^

Item	Control diet	10% CFP	20% CFP	SEM	*P* value
Microbial protein, mg/g	2.87	3.74	3.43	0.34	0.199
Volatile fatty acids, μmol/g					
Acetate	291.79	279.63	259.89	29.73	0.160
Propionate	109.31	105.35	88.81	9.92	0.299
Butyrate	66.20	58.38	46.28	8.30	0.265
Isobutyrate	14.34	12.11	11.36	1.18	0.205
Isovalerate	23.62	18.61	20.51	2.05	0.221
Valerate	23.75	22.70	19.76	2.73	0.551
Ammonium (NH_4_^+^), µmol/g dry matter	139.24	98.00	128.31	13.69	0.123

^1^CFP, corn fermented protein (Green Plains Energy, Omaha, NE, USA)

^2^Data are least square means of 8 observations per treatment

## Discussion

Historically, animal proteins have been used in weanling pig diets, but due to high costs and reduced availability of these ingredients, there is a need to identify other sources of AA that can be used in diets for weanling pigs. New technologies in the corn ethanol industry allow for removing some of the fiber in corn before fermentation, which results in a co-product with a greater AA concentration [3, 14, 15]. One of these new products is CFP, which contains around 50% CP, and CFP may, therefore, be included in diets for weanling pigs [3]. However, reduced growth performance by pigs fed CFP or other corn proteins has been reported [3, 16]. The reason for the reduced growth performance may be the high concentration of Leu in corn protein because excess Leu in diets for growing pigs influences the metabolism of Val and Ile by increasing Val and Ile catabolism, resulting in a deficiency of Val and Ile for protein synthesis [6]. This is because Leu, Val, and Ile share the first two steps in their catabolism. In the first step, there is a reversible transamination of Leu, Val, and Ile by branched-chain amino acid aminotransferase, which produces α-keto isovalerate, α-ketoβ-methylvalerate, and α-ketoisocaproate, respectively [17]. The second step is an irreversible decarboxylation of the keto acids by the branched-chain α-keto acid dehydrogenase complex [18, 19]. However, if there is an oversupply of Leu, there will be a greater concentration of α-ketoisocaproate, which stimulates the branched-chain amino acid aminotransferase enzyme and the branched-chain α-keto acid dehydrogenase complex. Therefore, excess dietary Leu increases the catabolism of Val and Ile [20] causing an AA imbalance [5], which results in reduced growth performance of pigs [5, 21]. Excess dietary Leu is also detrimental to the synthesis of serotonin, which is a neurotransmitter that influences feed intake regulation [7]. Serotonin is synthesized in the brain from Trp, but Trp and Leu share the same transporter into the brain and excess dietary Leu, therefore, may prevent Trp from being transported into the brain by occupying the transporter [5]. Reduced serotonin synthesis results in reduced feed intake and pigs with reduced feed intake due to excess Leu also have reduced growth performance [4, 5].

To prevent the negative effects of excess Leu in the present experiment, 0.10% extra Val or Ile or 0.05% extra Trp was added to diets containing CFP, becase these levels of inclusion have been demonstrated to miticate the negative impact of excess Leu in diets for growing pigs [7]. Diet analyses confirmed that all diets contained the intended concentrations of all AA. The control diets without CFP had calculated Val:Leu ratios of 0.59:1 and 0.63:1 in Phases 1 and 2, respectively, and the Ile:Leu ratios in the control diets were 0.55:1 and 0.53:1 in Phase 1 and Phase 2 diets, respectively. However, in the diet with 20% CFP and no added AA, the Val:Leu ratio was 0.46:1 and the Ile:Leu ratio was 0.38:1 in both Phase 1 and Phase 2 diets. By adding 0.10% crystalline Val and 0.10% crystalline Ile to the CFP diet, the Val:Leu ratio was increased to 0.52:1 in Phase 1 and to 0.51:1 in Phase 2, whereas the Ile:Leu ratio was increased to 0.43:1 in Phase 1 and to 0.42:1 in Phase 2. The chosen additions of crystalline Val and Ile, therefore, did not bring the Val:Leu and the Ile:Leu ratios back to the same levels as in the control diet and it is, therefore, possible that an additional increase in growth performance can be obtained if greater inclusions of Val and Ile are used.

The observation that the final BW, ADG, and ADFI decreased as the CFP without AA supplementation increased in the basal diets is in agreement with data indicating that 20% or 30% high-protein distillers dried grains with solubles resulted in a reduced growth performance of pigs [16]. Likewise, weanling pigs fed up to 10% CFP at the expense of enzyme-treated SBM had reduced BW, ADG, and G:F [22]. In contrast, CFP was also included in diets for weanling pigs by up to 10% without negative effects on growth performance [3], and up to 14% CFP was used in the first two phases after weaning without adversely affecting final BW, ADG, or ADFI [23]. Thus, responses to inclusion of CFP in diets for weanling pigs have been inconsistent, which may be a result of different degrees of imbalances among the branched chained AA depending on diet composition. Indeed, there was a negative correlation between dietary Leu levels and growth performance of pigs, whereas increased ratios between Val and Ile to Leu were positively correlated with growth [24].

In the current experiment, the objective was to determine effects of extra Val, Ile, and Trp, independently or in combination, on growth performance of pigs fed diets with excess dietary Leu. The observation that the reduction in final BW and ADG that was observed in pigs fed the diet with 20% CPP could be overcome if the 20% CFP diet was supplemented with Val, Ile, and Trp supports the hypothesis that requirements for all three AA are increased in high Leu diets. Likewise, the observation that addition of Val alone or in combination with Trp or Ile resulted in ADG that was greater than if Val was not used indicates that Val was the first limiting AA in the diets. However, because addition of Val alone was not sufficient to fully restore growth performance, it is concluded that requirements for Ile and Trp were also increased in the high Leu diets. Increasing ADG of pigs fed high Leu diets by increasing dietary Val has been demonstrated previously [25, 26]. The observation that effects of adding only Trp was limited is also in agreement with previous data [27]. This may be because only 3% of dietary Trp is used for serotonin synthesis [28], and in rats fed excess Leu, it was necessary to supplement Trp in combination with Val and Ile to overcome a reduction in growth performance [29].

The observation that there is no added advantage of adding Ile and Val compared with only Val is in agreement with data demonstrating that Ile is less able to counteract the negative effects of excess Leu than Val [24]. This is also in agreement with data demonstrating that Ile supplementation only partially overcame the negative effects of excess Leu [30]. This may be because the uptake competition for Leu is greater for Ile than for Val [27], which may be because the Km of the neutral AA transporter is highest for Val, followed by Leu and Ile [31].

The observation that pigs fed the diet containing 20% CFP and Val, Ile, and Trp had growth performance that was not different from that of pigs fed the control diet indicates that the negative effects of high-Leu diets can be overcome if CFP-diets are fortified with crystalline Val, Ile, and Trp. Infusion of Leu in neonatal pigs depleted plasma concentrations of Val and Ile to a greater extent than the depletion of other AA [32], which possibly is a consequence of the increased metabolism of not only Leu, but also of Ile and Val, if Leu is in excess. It was, therefore, demonstrated that to maximize protein synthesis, additional AA needed to be provided [32]. These observations are in agreement with data demonstrating that the optimal SID Ile to Lys ratio increased as Leu:Lys ratio increased in diets for 8 to 25 kg pigs [33]. Adding both Trp and Val to a diet high in Leu and fed to growing pigs may also prevent the detrimental effects of excess Leu on growth performance [7, 34]. As a consequence, the negative effects of an oversupply of Leu may be partially ameliorated by increasing dietary Trp, and both ADFI and ADG of growing pigs fed high Leu diets were increased by adding additional Trp to the diets [34]. However, reduced Trp concentration in the brain may not be the only reason for reduced ADFI of pigs fed high Leu diets. The mammalian target of rapamycin (mTOR) detects signals related to nutrients, energy, and hormones, influencing the regulation of metabolism and energy balance [35]. The activity of mTOR in the hypothalamus regulates feed intake [36]. Branched-chain amino acids among other AA are stimulators of mTOR even though the mechanism is not fully elucidated [35]. However, excess Leu can overstimulate mTOR, which suppresses feed intake, whereas excess Val does not have negative effects on feed intake [37], and inhibits the transport of Leu through the blood–brain barrier to a larger extend than Ile or Trp [38]. Increasing both Val and Ile in high Leu diets partially recovered the G:F ratio, but did not correct the reduced ADFI when compared with a control diet without inclusion of CFP [22], which further indicated that a part of the reduced ADFI that was observed in this experiment was caused by reduced availability of Trp to synthesize serotonin.

The observation that pigs fed diets with 20% CFP had improved fecal score compared with pigs fed the control diet is in agreement with data demonstrating softer feces from pigs fed a corn-SBM diet than pigs fed a diet with CFP [23]. The reason for these observations may be the presence of yeast in CFP because yeast may reduce diahrrea in pigs [39].

Blood urea N is positively correlated with urinary N excretion, and is, therefore, an indicator of AA utilization efficiency [40]. The observation that pigs fed the CFP diets with supplemented Val, Trp, and Ile had reduced blood urea N compared with pigs fed the control diet indicates that protein utilization in these pigs was more efficient. This also indicates that Val was the limiting AA in diets containing 20% CFP and diets containing more Val, therefore, likely had a more balanced AA composition with less excess AA compared with the control diet.

Albumin binds and transports nutrients in the blood [41]. Thus, the observation that there were only few differences in albumin concentration among treatments indicates that the reduced ADFI observed for pigs fed CFP diets did not impact the ability of albumin to transport nutrients. The lack of a difference in plasma immunoglobulin G among treatments indicates that the inclusion of CFP did not influence the immune response or the systemic health of pigs.

The protein in CFP is a combination of corn protein and protein from spent yeast because approximately 25% of the DM in CFP is spent yeast that contains 40% protein. The components of yeast cell walls (mannan oligosaccharides, nucleotides, and β-glucans) may provide health benefits when fed to animals [2], which is the reason the effect of CFP on gut health was determined. However, the lack of differences among treatments in immunoglobulin G indicates that under the conditions of this experiment, inclusion of CFP in the diets did not result in measurable improvements in immunity.

Changes in the intestinal morphological structure of weanling pigs, such as villus atrophy and crypt hyperplasia, is observed if pigs are fed diets that are inadequate in nutrient supply [42], due to a decrease in surface area, and a reduction in nutrient absorption. Thus, the lack of differences in morphology of pigs fed experimental diets indicates that inclusion of CFP in diets did not have any negative impacts on nutrient absorption of pigs when compared with pigs fed a diet based on SBM.

Distillers dried grains with solubles may increase growth of bacteria in the hindgut of pigs [43], and increased microbial growth may result in increased synthesis of VFA because of increased fermentation [44]. To the best of our knowledge, there are no data demonstrating effects of CFP on intestinal microbial protein, VFA, or ammonium in feces from pigs. However, the lack of a difference among treatments in microbial protein, VFA, and ammonium indicates that inclusion of CFP in diets for weanling pigs did not impact microbial growth and fermentation, which is likely due to the lower concentration of total dietary fiber in CFP compared with distillers dried grains with solubles.

## Conclusions

Inclusion of CFP in diets for weanling pigs improved fecal score in phase 2 and in most of the treatments with 20% CFP for the overall experiment. Inclusion of CFP in diets for weanling pigs did not affect immune system, ileum morphology, microbial protein, VFA, or fecal ammonium, but pigs fed a diet containing 20% CFP and added Val, Val and Trp, or Val, Trp, and Ile had a better protein utilization than pigs fed without those AA. However, using CFP in diets for weanling pigs without the use of supplementary AA depressed feed intake, and as a consequence, had a negative impact on growth performance, but this effect could be mostly overcome with supplementation of Val, Trp, and Ile. Therefore, up to 20% CFP may be included in diets for weanling pigs without affecting growth performance or blood characteristics, provided that provisions of Val and Ile are increased by 0.10% and Trp is increased by 0.05% compared with current requirement estimates. However, because the additions of crystalline AA used in this experiment did not bring the ratios back to the same as in the control diet, future research should be directed at determining the exact quantity of extra Val, Ile, and Trp that is required by pigs fed diets containing CFP.

## Data Availability

All data generated from the experiment are included in this article.

## Abbreviations

AA: Amino acids
ADFI: Average daily feed intake
ADG: Average daily gain
BW: Body weight
CFP: Corn fermented protein
CP: Crude protein
G:F: Gain-to-feed ratio
mTOR: Mammalian target of rapamycin
SBM: Soybean meal
VFA: Volatile fatty acids
